# Up-Date on Diabetic Nephropathy

**DOI:** 10.3390/life12081202

**Published:** 2022-08-08

**Authors:** Maria Chiara Pelle, Michele Provenzano, Marco Busutti, Clara Valentina Porcu, Isabella Zaffina, Lucia Stanga, Franco Arturi

**Affiliations:** 1 Department of Medical and Surgical Sciences, University “Magna Graecia” of Catanzaro, 88100 Catanzaro, Italy; 2 Nephrology, Dialysis and Renal Transplant Unit, IRCCS—Azienda Ospedaliero-Universitaria di Bologna, Alma Mater Studiorum University of Bologna, 40126 Bologna, Italy; 3 Oncology Unit, IRCCS—Azienda Ospedaliero-Universitaria di Bologna, Alma Mater Studiorum University of Bologna, 40126 Bologna, Italy; 4 Research Centre for the Prevention and Treatment of Metabolic Diseases (CR METDIS), University “Magna Graecia” of Catanzaro, 88100 Catanzaro, Italy

**Keywords:** diabetic nephropathy, hyperglycemia, type 2 diabetes, pathophysiology, albuminuria, therapeutics

## Abstract

Diabetes is one of the leading causes of kidney disease. Diabetic kidney disease (DKD) is a major cause of end-stage kidney disease (ESKD) worldwide, and it is linked to an increase in cardiovascular (CV) risk. Diabetic nephropathy (DN) increases morbidity and mortality among people living with diabetes. Risk factors for DN are chronic hyperglycemia and high blood pressure; the renin-angiotensin-aldosterone system blockade improves glomerular function and CV risk in these patients. Recently, new antidiabetic drugs, including sodium–glucose transport protein 2 inhibitors and glucagon-like peptide-1 agonists, have demonstrated additional contribution in delaying the progression of kidney disease and enhancing CV outcomes. The therapeutic goal is regression of albuminuria, but an atypical form of non-proteinuric diabetic nephropathy (NP-DN) is also described. In this review, we provide a state-of-the-art evaluation of current treatment strategies and promising emerging treatments.

## 1. Introduction

Diabetes Mellitus (DM) is defined by the presence of hyperglycemia due to a deficit of insulin production and/or resistance. Globally, 537 million adults between the ages of 20–79 years are diabetic, and the International Diabetes Federation (IDF) predicts a progressive increase in the number of diabetic patients up to 643 million by 2030 and to 784 million by 2045 [1]. DM is characterized by micro- and macrovascular complication; among the former, it is known that it causes up to 25–40% of chronic kidney disease (CKD), also called diabetic kidney disease (DKD), becoming the principal cause of kidney failure worldwide [2]. About one-third of patients with type 1 diabetes and about 50% of patients living with type 2 diabetes will develop CKD. According to Kidney Disease Improving Global Outcomes Work Group (KDIGO) guidelines, CKD is defined as a reduction of estimated glomerular filtration rate (eGFR) < 60 mL/min/1.73 m^2^ and/or presence of albuminuria. This classification includes patients who have increased albuminuria but normal eGFR (stage I and II) and those who have low eGFR with or without albuminuria (stage III, IV, and V). In fact, in large epidemiologic studies, many diabetic patients have an atypical form of non-proteinuric diabetic nephropathy (NP-DN) [3]. Risk factors for DKD are classified in two main groups: modifiable and non-modifiable ones. The former, susceptible to intervention, includes hypertension, hyperglycaemia, dyslipidaemia, and smoking. The second group comprises age, male sex, ethnicity, genetic factors (ACE, APOC1, APOE, HSPG2, eNOS, VEGF, TGFβ1, PPARγ, among others) [4]. Etiology of DKD includes multiple mechanisms such as glomerular hemodynamics (i.e., glomerular hyperfiltration), hyperinflammation, oxidative stress, and fibrosis. A large meta-analysis that includes about 1 million patients compared subjects with and without diabetes and demonstrated that patients with diabetes had a risk for all-cause and cardiovascular (CV) mortality up to two-fold more than those without. Owing to this evidence, several clinical trials have been conducted with the purpose of slowing the progression to ESKD and reducing kidney death and CV risk. In addition, these trials evaluated the nephroprotective role of drugs compared to the standard-of-care, often considered the renin-angiotensin-system inhibitors (RAASi). The SONAR, the FIDELIO-DKD, and CREDENCE trials demonstrated that endothelin-1 receptor antagonists (ERA), the novel non-steroidal mineralocorticoid receptor antagonist (MRA), and sodium-glucose co-transporter inhibitors (SGLT2is) improve renal outcomes in DKD patients already treated with RAASi. A personalized medicine is the best strategy to improve prognosis in DKD patients. In this review, we aim to conduct a state-of-the-art evaluation of current treatment and promising emerging treatments.

## 2. Diabetic Nephropathy: State of Art

“Diabetic nephropathy” (DN) refers to a well-defined kidney disease, directly associated with a long duration of diabetes and often confirmed by histological lesions [5]. DN is a heterogeneous disease characterized by the presence of albuminuria and/or reduction of eGFR in people with diabetes. According to the American Diabetes Association (ADA), pathological albuminuria is defined with an albumin-to-creatinine ratio (ACR) greater than 30 mg/g [6]. In type 1 diabetes mellitus (T1DM), it is rare to find DN in the first 10 years of disease, whereas the incidence of nephropathy increases between 10 and 20 years after the diagnosis (up to 3% per year). Although the duration of the disease is a strong predictor for the onset of DN, other factors are relevant for its development, including poor glycaemic control, uncontrolled blood pressure, and genetic susceptibility [7]. In T1DM patients, natural evolution of DN consists of glomerular hyperfiltration and evolves step-by-step with the onset of microalbuminuria, macroalbuminuria, and with the reduction of glomerular filtration rate (GFR) at the latest stages of the disease [3]. In type 2 diabetes mellitus (T2DM), most cases of DN do not rigidly follow the Mogensen’s phases, depicted in 1980; in fact, there is a greater heterogeneity in clinical presentation and according to different ethnicities. A large cohort study showed that the prevalence of albuminuria was higher in the Asian and Hispanic group (55%) than in Caucasian group (40,6%), likely due to polygenic component [8]. In the United Kingdom Prospective Diabetes Study (UKPDS), a large longitudinal cohort study, about one-third of diabetic patients developed kidney injury, detected with the presence of eGFR reduction or albuminuria. At time of diagnosis of T2DM, up to 3% of patients have already developed albuminuria, because frequently initial stages move on undiagnosed or as prediabetes [9]. In a third of cases, retinopathy is not present, unlike T1DM [10]. Albuminuria and co-existing retinopathy in T2DM are reliable markers of microvascular damage and thus highly suggestive of DN [11]. Presence of albuminuria often lead to the eGFR reduction and, in a portion of cases, to end-stage kidney disease (ESKD) [12]. However, in T2DM patients, DN can occur also without retinopathy or albuminuria in a non-trivial number of cases, and this can make it difficult to establish the correct timing to initiate the appropriate therapeutic intervention, mainly represented by RAASi, SGLT2 inhibitors, and other effective nephroprotective treatments [13].

**Figure 1:**
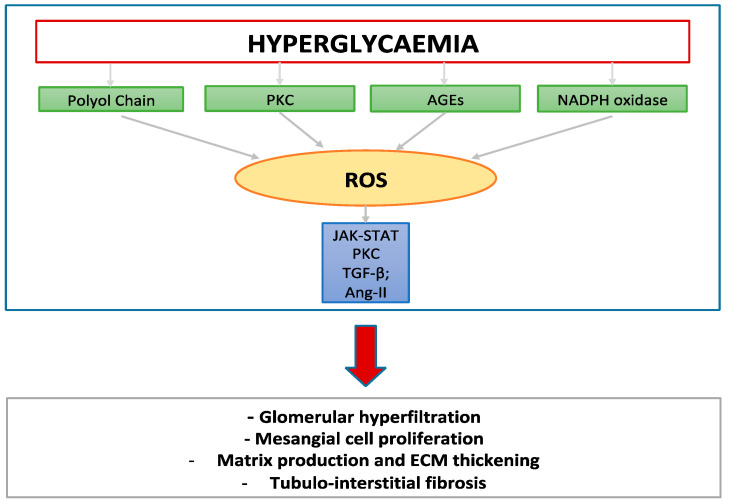
Mechanisms involved in the pathogenesis of diabetic kidney disease. PKC, protein kinase C; AGE, advanced glycated end-product; ROS, reactive oxygen species; TGF-β, transforming growth factor-β; Ang, angiotensin.

The pathogenesis of DN is complex and involves a number of mechanisms (Figure 1). Hyperglycemia is central in the pathogenesis of DN via different pathways. Hyperglycemia promotes the shunt of glucose metabolism toward non-glycolytic mechanisms such as the polyol pathway, which leads to the increased production of reactive oxygen species (ROS) [14]. The related oxidative stress increases local and systemic inflammation [15]. Moreover, oxidative stress is responsible for both direct and indirect damage to kidney cells (podocytes, mesangial, and endothelial cells). These cells are crucial for the modulation of glomerular and the filtration glomerular capillary structure. Hyperglycemia is associated with hypertrophy and mesangial cell proliferation, matrix production, and basement membrane thickening. Detrimental effects in the kidney cells determine proteinuria and tubule-interstitial damage and fibrosis [16]. Hyperglycemia is known to be responsible for generation of advanced glycation end-products (AGEs), which can alter the function and structure of the kidneys and initiate morphological changes typical of DN, leading to damage of the matrix, glomerular basement membrane, and other components in the glomerulus. Hyperglycemia also activates protein kinase C (PKC) pathway with production of endothelin-1 and vascular endothelial growth factor, determining glomerular damage and the direct oxidization of pivotal structures such as DNA, carbohydrates, lipids, and proteins. ROS can induce increased PKC activation, transforming growth factor -β (TGF-β) expression and angiotensin–II (Ang-II) levels [17], promoting fibrotic processes in the tubule-interstitium and remodeling of the extracellular matrix in the mesangium [14]. Moreover, Ang-II determines several effects on the kidneys, mediated by local and systemic activation of the RAAS [18]. Hyperactivation of Ang-II causes proteinuria, increases glomerular capillary pressure and permeability, and promotes inflammation and macrophage infiltration, leading to the production of inflammatory and profibrotic cytokines and extracellular matrix (ECM) remodeling. Moreover, it stimulates renal cell proliferation and hypertrophy [19,20]. In the progression of renal disease, RAAS plays a pivotal role [21,22]. The RAAS can be distinguished into a systemic and intra-renal RAAS and both of them are implicated in the pathogenesis of DN lesions. Systemic RAAS is a cascade of molecules that starts from the synthesis and secretion of renin from the juxtaglomerular cells in the kidney [23]. Renin cleaves angiotensinogen (AGN) producing angiotensin I (AGTI). Angiotensin I is then converted into angiotensin II (AGTII) via the angiotensin converting enzyme (ACE). AGTII is the main effector of the RAAS and acts through angiotensin receptors AT1 and AT2. A paracrine role of AGTII has been postulated and proved with the discovery of high concentration of intratubular and interstitial AGTI and AGTII. It has recently been demonstrated that renin may act independently of the generation of AGTII, confirming the existence of an intrarenal RAAS [24]. Several studies have also shown a reduction in circulating AGTII levels in diabetic patients, suggesting that the effect of AGTII on accelerating the progression of kidney disease is mediated by paracrine mechanisms in the kidney. AT1 receptor is widely expressed across the kidney tubules, glomerular wall, and arterial vasculature. These receptors have a well-known vasoconstrictive effect on systemic circulation and stimulate the secretion of aldosterone. However, additional intrarenal functions, such as arterial vasoconstriction, increase in sodium reabsorption across the tubules and regulation of tubule-glomerular feedback have been detected [20]. AT2 receptors are mainly expressed during embryogenesis. However, in keeping with recent experiments, it looks like moderate expression of AT2 receptors is maintained in the adult kidney where they counteract the actions of AT1 receptors. This is especially done through the stimulation of bradykinin, which in turn acts as vasodilator and natriuretic peptide. At least part of the beneficial effects of angiotensin receptor antagonists is due to the stimulation of AT2 receptors [25]. In patients with diabetes, AGTII is recognized as a main mediator of chronic injury. A crucial point of pathophysiology in patients with diabetes is the intrarenal activation of RAAS associated with an increased sensitivity to AGTII [26]. The RAAS inhibition proved a benefit in patients with DN, by delaying worsening of kidney function over time. However, the regulation of RAAS is complex and still not completely understood. The intrarenal levels of renin as well as the upregulation of renin RNA in proximal are increased in the early phases of diabetic nephropathy [27]. Moreover, the presence of hyperglycemia stimulates AGT mRNA expression in the proximal tubule of the kidney, and this increased gene expression is likely mediated by the reactive oxygen species (ROS) [28]. Hyperglycemia increases the production of superoxide in mitochondria, which, in combination with NO, generates peroxynitrite, a marker of vascular complications in diabetes. These findings are accompanied by the discovery of a downregulation of AT2 receptors in DN whose activation prevents the worsening of kidney damage in response to RAAS blockade drugs [29]. The resulting increase in AGTII intrarenal levels are deleterious on the kidney. Both hyperglycemia and AGTII activate the TGF-β pathway and promote extracellular matrix deposition with accumulation of collagen and fibrosis over time [30]. Interestingly, SGLT2 co-transporter contributes to the generation of ROS [31]. SGLT2 is hyperactivated in patients with DN, leading to an increase in sodium reabsorption in the proximal tubule. This mechanism is associated with the molecular dysfunction of other transporters and the energy consumption used to warrant the movement of sodium through the cell membranes. The resulting increased mitochondrial phosphorylation leads to a raised production of ROS.

SGLT2 cotransporter expression in the apical membrane of proximal tubular cells is pivotal in the development and progression of DN. Expression is regulated by levels of blood glucose. Hyperglycemia leads to an increased expression of SGLT2 [32]. AGTII intrarenal levels increase the expression of SGLT2 with a mechanism that involves AT1 receptors [33]. Other factors are involved in the regulation of SLGT2 expression including hepatocyte nuclear factors HNF-1α and HNF-3β [34]. The overall regulation of SGLT2 expression should be further clarified by confirming the importance and interconnection between multiple pathways (e.g., AGTII, SGLT2, ROS) in the pathogenesis of DN.

Additionally, aldosterone is involved in the pathogenesis of DN. Through the activation of the Smad2-dependent TGFβ1 pathway, aldosterone increases production of the extracellular matrix protein fibronectin, by glomerular mesangial cells [35]. Moreover, aldosterone increases collagen deposition leading to matrix and tubulointerstitial fibrosis, via ERK1/2-dependent pathways [36]. Hyperglycemia stimulates renin and Ang-II synthesis in mesangial cells [37]. Much evidence supports the role of interleukins (such as IL- 1, IL-18, and IL-6) in the progression of DN [38]. Plasma levels of these cytokines increase with the development of nephropathy and are independently associated with the development of albuminuria [38]. In experimental DN models, inhibition of inflammation state has been shown to have a protective effect on the kidney [39]. Nuclear factor-κB (NF-κB) is one of the key elements involved in the inflammatory process of DN, regulating cell adhesion proteins, inflammatory cytokines, and chemokines, ultimately contributing to kidney injury [40]. Cytokines and hyperglycemia can activate important pathways, such as the Janus kinase/signal transducers and activators of transcription (JAK-STAT) pathway, which is the main mediator between paracrine stimulation and nuclear receptors. [41]. It is upregulated in glomerular cells in early DN and promotes NF-κB. It is activated by several stimuli, such us hyperglycemia, mechanical stress, AGE and ROS, Ang-II, inflammatory cytokines, and leads NF-κB to stimulate the production of proinflammatory cytokines and adhesion molecules, establishing a vicious circle [40]. Another important pathway activated in DN is intraglomerular hypertension, induced by glomerular hyperfiltration. It is one of the first mechanisms responsible for the onset of albuminuria and eGFR reduction and is mediated by glucose-dependent dilation of glomerular afferent arteries, through vasoactive mediators such as TGF-β1, vascular endothelial growth factor (VEGF), insulin-like growth factor 1 (IGF-1), nitric oxide (NO), prostaglandins, and glucagon [42]. Kidney hypoxia is a crucial factor that promotes progression of DN. In diabetic people, hyperglycaemia increases energy consumption of tubular cells, due to glomerular hyperfiltration and upregulation of sodium–glucose cotransport [43]; moreover, loss of peritubular capillaries and interstitial fibrosis decreases oxygen delivery [44], causing a mismatch between oxygen demand and supply. Recent studies have also demonstrated that dysregulated autophagy is important in physiopathology of DN; the mechanism is not fully understood and seems to play a crucial role the mammalian target of rapamycin complex 1 (mTORC1), which negatively regulates autophagy by inhibiting the activity of Unc-51-like kinase 1 (ULK1), a kinase important for a start of the autophagy [45]. Animal and human studies demonstrated that inhibition of mTORC1 by rapamycin decreases the progression of DN in diabetic db/db mice [46]. Future studies should address in more detail the interconnections between the different pathways of damage in DN and the potential molecular target for novel pharmacological drugs, which are eagerly expected in the context of this severe disease.

## 3. DN and Risk for Progression to ESKD—Novel Biomarkers

Patients with DN have a high risk for progression to end-stage kidney disease (ESKD). In addition, they have also a very poor CV prognosis. The Alberta Kidney Disease Network (AKDN) cohort and National Health and Nutrition Examination Survey (NHANES) showed that patients with DN had a 2.7 times higher risk of acute myocardial infarction [45]. This risk was higher than the one presented in patients with diabetes (2.0) or CKD (1.4) alone. Moreover, they also reported higher risk for all-cause mortality. Blood pressure, age, serum lipids levels, and smoking habit play an important role in CV events in DN patients [46]. However, hyperglycemia is a modifiable risk factor such as hypertension and other metabolic factors. Recent studies have showed that an equally remarkable role should be given to proteinuria (or albuminuria) and eGFR reduction, non-traditional risk factors [47]. In diabetic patients, proteinuria acts as a CV risk modulator. Data of a cohort of CKD subjects showed that the risk for major CV events (MACE) starts for mild–moderate 24 h-proteinuria (0.150–0.500 g/24 h) in DN subjects, compared with CKD subjects without T2DM where the risk starts from the severe proteinuria category (>0.500 g/24 h). Historically, albuminuria is also considered as a biomarker of progression of DN and a marker of glomerular damage, although the albuminuria classification does not include all the mechanisms and risk factors that are present in subjects with DN. Recently, clinical research has focused on finding novel biomarkers. Symmetric Dimethylarginine (SDMA) is a catabolic product of arginine-methylated proteins, excreted through the kidneys. A study from Zobel et al. showed that SDMA is increased in patients with T2DM and microalbuminuria and is associated with impaired renal function and CV disease [47]. Looker et al. demonstrated that SDMA is associated with proteinuria and inversely associated with GFR in subjects with T2DM and DN, and SDMA to asymmetric dimethylarginine (ADMA) ratio predicts renal function decline [48]. Another study showed that SDMA, in T1DM patients with microalbuminuria and high eGFR, is lower than the group with normoalbuminuria [49], perhaps due to hyperfiltration. Another marker of renal damage is cystatin C; it is freely filtered by the glomerulus and inversely correlated with eGFR [50]. Interestingly, cystatin C is less influenced by muscle mass than creatinine and it has lower inter-individual variability [51]. Blood levels of cystatin C is affected by thyroid disorders or glucocorticoid treatment [52]. In patients with T2DM, the concentration of cystatin C is independently associated with GFR, and its increasing is observed in patients with normoalbuminuria and decreased GFR [53]. Another tubular damage marker is retinol-binding protein-4 (RBP-4), a carrier of retinol in plasma that is not reabsorbed by tubular when it is damaged, so its urinary concentration can be considered a predictive marker of DN in diabetic and macroalbuminuric patients [54,55]. Although the plasma concentration of RBP-4 is not a better marker of serum cystatin C or creatinine [56], in humans and animal models it is a marker of CV risk factors and insulin resistance [57,58,59]. In survival models, plasma levels of tumor necrosis factor receptors (TNFR-1 and TNFR-2) are linked with an enhanced risk of CKD progression and ESKD. Furthermore, they may help to ameliorate risk stratification of DN subjects [60]. Interestingly, these two biomarkers predict ESKD independently with proteinuria, so they may play a possible predictive role in the earlier stages of DN and in non-proteinuric phenotypes of DN. It has been hypothesized that TNFR-1 and TNFR-2 have a direct toxic effect on the kidney, activating pathways of inflammation and apoptosis. Neutrophil gelatinase-associated lipocalin (NGAL) is produced in the renal tubule due to inflammation injury [60]; in T2DM patients it is inversely related with eGFR, but positively related with albuminuria [61,62]. In normoalbuminuric diabetic subjects compared to non-diabetic control subjects, NGAL is higher [63]. Furthermore, urinary NGAL levels are higher in T2DM diabetic patients with hyperfiltration compared to T2DM with normal eGFR [64]. It must be said that NGAL levels could be affected by urinary tract infections, obstructive pulmonary disease, different types of neoplasms, and preeclampsia [65]. Kidney Injury Molecule -1 (KIM-1) is a type 1 transmembrane glycoprotein located in the proximal tubules, proposed as a marker of acute kidney injury; high concentration in T2DM patients with normal to mild albuminuria have been reported [66]. Two studies found that the use of KIM-1 associated with pro b-type natriuretic peptide (pro-BNP) or beta 2 microglobulin, GFR, and albuminuria in T2DM seems to improve prediction of kidney function decline [67,68]. Another study showed that KIM-1 is not associated with albuminuria; moreover, in a study of T1DM patients, it does not seem like it is a predictor for progression of DN [69]. it should be noted that urinary tract disease and sepsis can enhance urinary KIM-1 [70]. The high-sensitivity cardiac troponins (hs-cTnT and hs-cTnI) and the NT-proBNP are currently used for diagnosis of acute coronary syndrome and heart failure, respectively. In patients with kidney disease, the dosage of troponins improves CV risk stratification. Similarly, NT-proBNP has been shown to predict CV and kidney outcomes in subjects with kidney disease. Transforming growth factor-b1 (TGFb1) is a cytokine and a mediator of kidney damage, leading to mesangial matrix expansion, interstitial fibrosis, and glomerular membrane thickening ultimately leading to a kidney injury and reduction in GFR [71]. In animals’ models, TGFb1 participates in podocytes injury and apoptosis, stimulating expression of VEGF, which increases collagen deposition in basement membrane [72]. In in vitro studies, TGFβ1 seems to lead oxidative stress in podocytes by itself, and podocyte injury leads to proteinuria [73,74]. Furthermore, in vitro studies showed that high glucose concentration stimulates TGFβ1 secretion and activation, so it is proposed as mediator of DN in animal models [75,76]. A systematic review, which included a large number of T2DM patients, showed a positive correlation with high levels of serum and urinary TGFβ1with albuminuria [77]. Treatment with angiotensin-converting enzyme inhibitors (ACE-i) decreased levels of TGFb1, which could be a mechanism of nephro-protection [78]. VEGF is an important angiogenic factor [79]. In the kidney, reduction of oxygen delivery is a stimulus of expression of VEGF [80,81]; studies in vitro demonstrated that chronic hyperglycemia can enhance the production of the VEGF protein [82]. In diabetic humans’ biopsies, a down regulation of VEGF-A expression is found that is correlated with loss of podocytes [83]. Moreover, in people with T1DM and T2DM and advanced DN, higher levels of urinary VEGF have been described [80]. There is contradictory evidence in interventional studies, where both inhibition and administration of VEGF improve kidney function [84]. Soluble urokinase-type plasminogen activator receptor (suPAR) is the circulating form of membrane protein urokinase receptor (uPAR); both regulate cell adhesion and migration [85]. Their increased level is an independent risk factor of CV diseases and kidney disease. Some evidence indicated suPAR such as a marker of early kidney disease. In a cohort study that included subjects with T1DM, suPAR levels correlated with decline in eGFR and CV risk, but not with albuminuria [86]. Conversely, other studies demonstrated a positive correlation in T1DM and T2DM patients between levels of suPAR and albuminuria [87,88]. Growth differentiation factor-15 (GDF-15) is a cytokine, that is a marker of inflammation and of oxidative stress [89]. In people with diabetes, GDF-15 seems to be a marker of impaired fasting glucose, and its higher levels are correlated with an increased risk for several adverse outcomes, such as progression of albuminuria in T2DM patients [90], eGFR decline and CV risk in T1DM patients [91], and early death in patients in haemodialysis [92], and it is associated with heart failure in general population [93]. In a post hoc analysis of the CANVAS trial, authors found that increased plasma levels of GDF-15 were associated with 20 to 30% higher risk for CV events, 1.5 to 2.1 higher risk for the development of heart failure and up to three-fold higher risk for kidney outcomes, respectively; results were adjusted for albuminuria, eGFR. Administration of canagliflozin decreased the mean level of GDF-15 as compared to levels of start-of-treatment visit; instead, the treatment effect on future outcomes did not depend on the GDF-15. Matrix metalloproteinases–10 (MMP-10) is involved in kidney and CV disease [94]. In fact, evidence in patients with CKD showed that elevated concentrations of MMP-10 are independently associated with atherosclerosis and, in T1DM patients, are associated with DN [95,96]. Conversely, it is not associated with eGFR decline [97]. MMP-10 remodels matrix, and its degradation products promote mesangium expansion, which leads to nephropathy, so MMP-10 has been suggested as a reliable drug target to attenuate the progression of retinopathy and DN [98]. Risk stratification is crucial in patients with DN since not all patients within the same stage of disease have the same prognosis. Hence, novel biomarkers would be helpful in the future to better classify DN patients according to their future prognosis and to guide clinicians in assessing the correct clinical management (Table 1).

## 4. Old and New Drugs Capable of Ameliorating Risk in Patients with DN

One of the first studies in subjects with T2DM and nephropathy dates back to early 2000 [98]. Three main studies, the Collaborative Study Group (CSG) Captopril trial, the IDNT, and the RENAAL trial, have demonstrated the safety and efficacy of angiotensin-converting-enzyme inhibitors (ACEi) and Angiotensin Receptor Blockers (ARBs) in decelerating CKD progression in subjects with DN [98,99,100]. On average, ACEi and ARBs led to the reduction of about 20% of the risk of ESKD in comparison with standard-of-care. According to this important evidence, treatment with RAASi has grown rapidly, although post hoc analyses of these trials have showed that up to 40% of subjects were not responders to ACEi or ARBs in term of albuminuria reduction [101,102]. This variability in efficacy (called true variability or non-random variation) is independent of daily fluctuations (random variation) in albuminuria and is partially explainable by several factors such as albuminuria level at the moment of start-of-treatment with RAASi and adherence to treatment [103]. Even more importantly, the variability in albuminuria response is reproducible over time, namely when the same drug is withdrawn and then restarted, or when other treatments belonging to the same drug class are initiated. Moreover, variability in response to treatment with RAASi was also demonstrated for secondary targets of response (off-targets) such as uric acid, hemoglobin, and serum potassium levels [104]. Regardless of the cause of variability, the clinical implication of these findings is that there is a high residual risk in DN patients due to the persistence of albuminuria. For this reason, several trials have been conducted in the past two decades with the purpose of decreasing this residual cardiorenal risk in patients with DN. In these trials, different combinations of treatments have been evaluated, namely dual RAAS blockade (Aliskiren + ARB/ACE in the ALTITUDE trial; ACEi + ARB in the VA-NEPHRON-D trial), antioxidants (Bardoxolone, BEACON), endothelin receptor antagonist (avosentan in the ASCEND trial) [105,106,107,108]. Unfortunately, most of these trials failed to show a significant effect of treatment, or even reported a raised CV risk in the treatment arm of the study leading to an earlier interruption of the study itself. Two phenomena could explain these findings: (1) the ‘add-on’ effect, which consists of adding a treatment to a drug of the same class, which did not determine a significant response; (2) the study design of clinical trials in the past. In fact, in all the mentioned trials, patients were randomized to receive standard-of-care or the experimental (potentially useful) treatment without considering the likely of response to treatment in the two arms. Two large trials, carried out in DKD patients, were published in 2019 (two decades after the previous) [109,110]. These studies have highly found that SGLT2 inhibitors and selective ERA have an important role in slowing CKD progression. SGLT2 inhibitors are antagonists of the SGLT-2 co-transporter, present in the early proximal renal tubule, that is capable to reabsorb about the 90% of filtered glucose. In T2DM, SGLTs are overexpressed in the proximal tubules, due to hyperglycemia, and this determines an increase in Angiotensin II intrarenal synthesis. The overexpression of SGLT proteins and mRNA, also showed in tubular cells, increases glucose reabsorption, as observed in diabetic subjects. SGLT2 inhibitors increase glycosuria, and thus determine an amelioration of glycemic control. Furthermore, they also lead to a restoration of the normal tubule–glomerular feedback (TGF), which is linked to the long-term protection on the kidney and anti-fibrotic and anti-inflammatory effects, determining to a reduction of ROS production, tubule-interstitial fibrosis, and glomerulosclerosis. Recently, dapagliflozin, an SGLT2i, has been demonstrated to reduce the renal resistive index [111,112], with a potential enhancement of endothelial function in the kidney. The CREDENCE trial enrolled more the 4000 patients with DKD who were randomized to standard-of-care (one ACEi or ARB) or the SGLT2i canagliflozin (added to standard-of-care) [110]. This large trial reported that canagliflozin warrants a 30% lower risk of renal events (ESKD, doubling of serum creatinine and death from renal or CV causes) when added to a RAASi. Intriguingly, a significant risk reduction was observed in this study also in respect to secondary outcomes, which included CV death, myocardial infarction, and hospitalization for heart failure. The CV protection of SGLT2 inhibitors was confirmed in the Canagliflozin Cardiovascular Assessment Study (CANVAS) trial [113]. Over 10,000 patients with T2DM and high CV risk, treated with canagliflozin, presented a 15% reduction in the risk of fatal and non-fatal CV events. Another drug class interesting for the treatment of DN is represented by the ERA. ERA are selective antagonists of endothelin-1 receptor A, which are capable of activation that has been associated with the development of glomerulosclerosis and albuminuria in conditions of increased production of endothelin-1, as it presents in CKD subjects. In addition, endothelin-1 binding to ET receptors type A (ETAr), promoting cell proliferation, oxidative stress, inflammation, podocyte activation, vasoconstriction, and stimulation of angiotensin II, all mechanisms that worsen the decline of renal function [114]. Although the negative results were achieved with avosentan [108], the more selective atrasentan has shown, in the SONAR trial, to lead a reduction the risk of renal events (i.e., ESKD or doubling of serum creatinine) by 35%, in addition to RAASi treatment [109]. Moreover, atrasentan was associated with hypervolemia due to fluid retention, as demonstrated by the increasing Brain-Natriuretic-Peptide (BNP) levels compared with placebo group. In the FIDELIO-DKD trial [115], about 5700 subjects with DN were treated with the steroidal MRA finerenone compared to standard-of-care (RAASi). The conclusions are very interesting: finerenone group had a 20% lower risk of developing renal events. Moreover, the non-steroidal MRA, like the steroidal MRA, such as Spironolactone and Eplerenone, block the binding of aldosterone to its receptors, leading to a degradation of ENaC channels and then natriuresis. This novel drug class is advantageous thanks to its greater affinity and selectivity for the mineralocorticoid receptor and a low risk of serious adverse events, (i.e., hyperkalemia, gynecomastia, and decline of kidney function). None of these studies have shown that the combination of MRA, ERA, and SGLT2 can reduce the residual risk of CKD progression or in which patients this combination could induce nephroprotection. It is really interesting, however, that these drugs decrease the excretion of urine albumin, reducing or abolishing their adverse events each other. For example, the natriuretic effect of SLGT2is mitigates the fluid retention associated to ERA, through the endothelin receptor B and the hyperkalemia linked to MRAs. Another drug class that conferred a positive result in DKD patients were glucagon-like peptide-1 receptor agonists (GLP1-RA) [116]. These agents stimulate the incretin GLP1 receptors and thus reduce glucagon release and stimulate insulin secretion from pancreatic β-cells [117]. The AWARD-7 trial [118] evaluated the safety and efficacy of the GLP1-RA dulaglutide in subjects with DKD and demonstrated that treatment with dulaglutide, at both doses of 0.75 mg and 1.5 mg per day, was associated with a minor eGFR decline as compared to insulin glargine. The aim of the AMPLITUDE-O trial was to compare CV and kidney (decrease of eGFR or increase in albuminuria) outcomes in diabetic subjects, randomized to receive efpeglenatide or placebo, with previous history of CV disease or current presence of CKD [119]. In the efpeglenatide group, risk for the onset of both CV and kidney events was 30% lower than the control group. The above-mentioned studies reported kidney and CV protection, above all in the patients that manifest a positive response in the first months of treatment, regardingHbA1c, albuminuria and blood pressure reduction. Moreover, a post hoc analysis of the RENAAL trial emphasized that, in the losartan group, the importance of nephroprotection, in term of ESKD risk reduction, was directly proportional to the degree of albuminuria reduction, early after treatment initiation [120]. The same findings were found in the ALTITUDE trial, although it was a ‘negative’ study. In fact, in this study, subjects randomized to receive aliskiren + ACE/ARB had less than half the risk of CKD progression as compared with those that received ACE/ARB alone [121]. A significant learning from these relevant studies is that within each treatment group there was a consistent variability in progression. The aim for next studies is to decrease variability in response to the well-known biomarkers. Interestingly, it has been well-highlighted that a non-negligible proportion of DN patients progress to the more advanced stages of CKD (3 to 5) although the ACE/ARB-induced albuminuria reduction [122,123]. This controversial context reveals that DN is a multifactorial and multi-marker-based disease, and that finding novel predictive and prognostic biomarkers is crucial. The principal findings of this study are summarized in Table 2. Despite the positive results of the more recent clinical trials regarding the treatment of DN, many patients remain at increased cardiovascular and kidney risk. The pharmacologic research should provide evidence on the efficacy of novel treatments that can reduce and minimize such large residual risk.

## 5. Conclusions

DN is a heterogeneous disease associated with an extremely high risk of CV events, CKD progression, disability, and all-cause mortality. The global burden is becoming more severe, as testified by the increasing of prevalence of diabetes worldwide. For this reason, there is a great interest in searching for prognostic biomarkers that are able to improve risk stratification of DN patients. In this context, predictive biomarkers are also expected in the perspective of precision medicine, being crucial to tailor pharmacological treatment to the individual risk profile. Recent studies have clarified some concepts regarding the comprehension of the mechanisms that influence the individual response to cardioprotective and nephroprotective treatments. In conclusion, it is desirable that positive evidence from clinical trials could be quickly transferred to clinical practice. Further studies about this interesting and important topic are more than expected in the near future.

## Figures and Tables

**Table 1:** Summary of the principal prognostic and predictive biomarkers in diabetic nephropathy.

Biomarkers	Characteristics	Prognostic/Predictive Values
SDMA	It is a catabolic product of arginine methylated proteins, excreted through the kidneys	Increased in patients with T2DM and microalbuminuria and is associated with impaired renal function and cardiovascular disease [34]. Is associated lower eGFR in patients with T2DM and DN; lower values have been observed in proteinuric patients perhaps due to hyperfiltration [35,36]
Cystatin C	It is a low molecular weight protein produced by all types of nucleated cells. It acts as inhibitor of cysteine protease and is freely filtered by the renal glomeruli, then 99% reabsorbed and metabolized in the renal proximal tube. It is not secreted. It is also a marker of tubular damage [38]	The concentration of cystatin C in T2DM patients is independently associated with eGFR, and its increasing is observed in patients with normoalbuminuria and decreased GFR [40]
RBP-4	It is a carrier of retinol in plasma, which is not reabsorbed by tubuli when they are damaged	It is a marker of tubular damage, and its urinary concentration can be considered a predictive marker of DN in diabetic and macroalbuminuric patients [41,42]. Although the plasma concentration of RBP-4 is not a better marker of cystitis C or creatinine [43], in humans and animal models it is a marker of insulin resistance and cardiovascular risk factors [44,45,46]
TNFR-1 and TNFR-2	They are membrane receptors that bind TNF alpha. It has been hypothesized that they have a direct toxic effect on the kidney, activating pathways of inflammation and apoptosis	Plasma levels of TNFR-1 and TNFR-2 are linked with an enhanced risk of CKD progression and ESKD. Moreover, they may help to ameliorate risk stratification of DKD patients [57]. They predict ESKD in absence of proteinuria, so they may play a possible predictive role in the earlier stages of DN and in non-proteinuric phenotypes of DN
NGAL	It is a protein produced in the renal tubule due to inflammation injury [47]	In T2DM patients, plasma levels of NGAL are inversely related to eGFR and positively related to albuminuria [48,49]. In normoalbuminuric patients with DM compared to non-diabetic control subjects, NGAL is higher [50]. Furthermore, NGAL urinary levels are higher in T2DM diabetic patients with hyperfiltration compared to T2DM with normal eGFR [51]
KIM-1	It is a type 1 transmembrane glycoprotein located in the proximal tubules, and it is proposed as a marker of acute kidney injury	In T2DM patients with normoalbuminuria or mild albuminuria, its plasma concentrations are high [53]. The use of KIM-1 associated with pro b-type natriuretic peptide (pro-BNP) or beta 2 microglobulin, eGFR, and albuminuria in T2DM seemed to improve prediction of kidney function decline [54,55]. It is not associated with albuminuria. Moreover, in T1DM patients, it does not seem like it is a predictor for progression of DN [56]
Cardiac troponins (hs-cTnT and hs-cTnI)	They are enzymes present in both skeletal and cardiac muscles. They regulated muscle contraction by controlling the calcium-mediated interaction of actin and myosin	Raises in their values are related to acute myocardial damage. In patients with kidney disease, the dosage of both hs-cTnT and hs-cTnI improves CV risk stratification
NT-proBNP	Amino terminal fragment of the natriuretic type B peptide, normally produced in the heart and released in the case of cardiac stresses consequent to water overload conditions	NT-proBNP has shown to predict CV and kidney outcomes in subjects with kidney disease
TGF β 1	TGF β 1 is a cytokine and a mediator of kidney damage, leading interstitial fibrosis, mesangial matrix expansion, and glomerular membrane thickening	TGFβ1 seems to cause oxidative stress in podocytes by itself, and podocyte injury leads proteinuria [60,61]. Moreover, in vitro studies showed that high glucose concentration stimulates TGFβ1 secretion and activation, so it is proposed as a mediator of DN in animal models [62,63]. A positive correlation of high levels of serum and urinary TGFβ1with albuminuria has been reported in a large metanalysis [64]
VEGF	VEGF is an important angiogenic factor [66]. In kidney, reduction of oxygen delivery is a stimulus of expression of VEGF [67,68]	Studies in vitro demonstrated that chronic hyperglycemia can increase the production of the VEGF protein [69], in diabetic humans biopsies, it is found a down regulation of VEGF-A expression that is correlated with loss of podocyte [70]. Moreover, in people with T1DM and T2DM and advanced DN, higher levels of urinary VEGF have been found. Although, there is contradictory evidence in intervention studies; in fact, both inhibition and administration of VEGF has been shown to improve kidney function [71]
suPAR	It is the circulating form of membrane protein urokinase receptor (uPAR), regulates both cell adhesion and migration [72]	Its increased levels are independent risk factors of cardiovascular diseases and kidney disease. Some evidence indicated suPAR as a marker of early kidney disease. In a cohort study that included patients with T1DM, suPAR is correlated with decline in eGFR and cardiovascular risk, but not with albuminuria [73]. Conversely, in another studies, it demonstrated a positive correlation in T1DM and T2DM patients between levels of suPAR and albuminuria [74,75]
GDF-15	It is a member of TGF-cytokine family, released in response to cellular stress. It seems to have a role in regulating inflammatory processes, apoptosis, cell repair, and cell growth [76]	Higher levels are correlated with an increased risk for several adverse outcomes, particularly to a progression of albuminuria in T2DM patients [77], eGFR decline and cardiovascular risk in T1DM patients [78], early death in patients in haemodialysis [79], and in the general population it is associated with incident heart failure [80]
MMP-10	It is a calcium-dependent endopeptidases that contains zinc, involved in the various processes of tissue development and cellular homeostasis. MMP-10 remodels matrix and its degradation products promote mesangium expansion [85]	It is involved in kidney and cardiovascular disease [81]. In patients with CKD, elevated concentrations of MMP-10 are independently associated with atherosclerosis and in T1DM patients are associated with DN [82,83]. Conversely, it is not associated with eGFR decline [84]

SDMA, Symmetric Dimethylarginine (SDMA); T2DM Type 2 diabetes mellitus; eGFR, estimated glomerular filtration rate; RBP-4 Retinol-Binding protein 4; TNFR 1 and TNFR 2, tumor necrosis factor receptors; NGAL, Neutrophil Gelatinase-Associated Lipocalin (NGAL); KIM-1, kidney injure moelcule-1; T1DM, Type 1 diabetes mellitus; NT-proBNP, N-terminal prohormone of brain natriuretic peptide; TGF β 1, transforming growth factor beta; VEGF, vascular endothelial growth factor; suPAR, soluble urokinase plasminogen activator receptor; GDF-15, growth differentiation factor 15; MMP-10, Matrix metalloproteinases-10; CKD, chronic kidney disease.

**Table 2:** Key messages provided by the present study.

	Key Points
	Diabetes is one of the most important causes of CKD. Diabetic kidney disease (DKD) is a major cause of ESKD worldwide, being associated with and increased CV and all-cause mortality risk.
DiabeticNephropaty	Risk factors for DKD are: chronic hyperglycemia, hypertension, proteinuria (or albuminuria), and eGFR.
	In diabetic patients, proteinuria acts like a CV risk modulator and is considered as biomarker of progression of DN and a marker of glomerular damage. Recently, clinical research has focused on finding new biomarkers.
	ACEi and ARB slow the CKD progression, but up to 40% of patients do not respond in term of albuminuria reduction.
	Sodium-glucose transport protein 2 inhibitors (SGLT2) and glucagon-like peptide-1 agonist have demonstrated additional contribution in slowing progression of kidney disease.
	Selective ERA confer a further 35% risk reduction of renal events added to RAASi but was also associated with increase of fluid retention and hypervolemia.

## Data Availability

Not applicable.

## Abbreviations

AGEs	Advanced glycation end-products
ADA	American Diabetes Association
Ang-II	Angiotensin –II
ACE-i	Angiotensin-converting enzyme inhibitors
ARBs	Angiotensin Receptor Blockers
ADMA	Asymmetric dimethylarginine
BNP	Brain-Natriuretic-Peptide
CV	Cardiovascular
CKD	Chronic Kidney Disease
DM	Diabetes Mellitus
DN	Diabetic nephropathy
DKD	Diabetic kidney disease
ESKD	End-stage-kidney-disease
ERA	Endothelin-1 receptor antagonists
ETAr	Endothelin-1 binding to ET receptors type A
eGFR	Estimated glomerular filtration rate
ECM	Extracellular matrix
KDIGO	Global Outcomes Work Group
GLP1-RA	Glucagon-like peptide-1 receptor agonists
GDF-15	Growth differentiation factor-15
hs-cTnT and hs-cTnI	High-sensitivity cardiac troponins
IGF-1	Insulin-like growth factor 1
JAK-STAT	Janus kinase/signal transducers and activators of transcription
KIM-1	Kidney Injury Molecule -1
mTORC1	Mammalian target of rapamycin complex 1
MACE	Major cardiovascular events
MMP-10	Matrix metalloproteinases—10
MRA	Mineralocorticoid receptor antagonist
NGAL	Neutrophil Gelatinase-Associated Lipocalin
NO	Nitric oxide
NP-DN	Non-proteinuric diabetic nephropathy
NF-κB	Nuclear factor-κB
PKC	Protein kinase C
uPAR	Protein urokinase receptor
ROS	Reactive oxygen species
RAASi	Renin-angiotensin-system inhibitors
RBP-4	Retinol-binding protein-4
SGLT2is	Sodium-glucose co-transporter inhibitors
suPAR	Soluble urokinase-type plasminogen activator receptor
SDMA	Symmetric Dimethylarginine
T1DM	Type 1 diabetes mellitus
T2DM	Type 2 diabetes mellitus
TGF-β1	Transforming growth factor—β1
TNFR-1 and TNFR-2	Tumor Necrosis Factors receptors
ULK1	Unc-51-like kinase 1
VEGF	Vascular endothelial growth factor
